# Surveillance and prayer – comparing Muslim prison chaplaincy in Germany’s federal states

**DOI:** 10.1186/s40878-017-0051-5

**Published:** 2017-03-16

**Authors:** Lisa Harms-Dalibon

**Affiliations:** Max Planck Institute for the Study of Religious and Ethnic Diversity, Göttingen, Germany

**Keywords:** Governance of Islam, Minority rights, Muslim chaplaincy, Religion in prison

## Abstract

Moving beyond approaches that emphasise the influence of national ideologies and transnational frameworks on the governance of religious diversity in Western Europe, recent scholarship has underlined the importance of analysing the impact of concrete institutional settings such as hospitals, schools and prisons on the public incorporation of religious minorities. Building on this approach, the present article analyses the emergence of Muslim prison chaplaincies in three German federal states by focussing on how framing strategies of state- and religious actors accommodate the national state-church framework and prison-related norms. The article thus shows how national ideologies of diversity regulation and prison norms are mutually shaped in the process of the local governance of Islam. The comparative perspective of the article highlights subnational variations regarding actor constellations and strategies and thereby emphasises a multidimensional process of negotiating the national regime of diversity governance.

## Introduction

With an increasingly diversified religious landscape in Europe, the accommodation of faith-related claims of minorities has come under a central spotlight of public debate and an important research concern. The relation between Western European states and Muslim communities in particular constitutes a topic of intense controversy. Certain religious symbols and practices, such as the headscarf, halal food, or the construction of minarets, have proven to be focal points for discussing state responses to Muslim demands for public recognition. A long-standing line of research has analysed the dynamics and outcomes of these controversies in a cross-national comparative fashion, highlighting differences in terms of national citizenship regimes and models of state-church relationships (Fetzer & Soper, 2004; Rath, Penninx, Groenendijk & Meyer, 2001) and similar trends of opening spaces for integrating minorities (Koenig, 2005; Koopmans, Michalowski, & Waibel, 2012). While this literature has produced important insights, recent scholarship has advocated disaggregating what often seem to be overly stylised national models (Bader, 2007; Bertossi, Duyvendak, & Van Reekum, 2012; Bowen, Bertossi, Duyvendak, & Krook, 2014). It argues that inclusion of religious newcomers should be analysed as a complex interplay between national-legal and political ideologies and concrete institutional rules, practices, and boundaries in settings such as schools, hospitals, or prisons. In fact, these locations are characterised, or so the argument goes, by distinctive institutional logics that may have a greater impact on practices of accommodation than do national ideological or legal frameworks. Following this approach, I have suggested in this article that both dynamics – national legal and institution specific – are actively shaped and articulated by collective actors negotiating the terms of diversity governance at the meso level.

To dissect the dynamic interaction of national and institution-specific settings, I have focused in this paper on Muslim chaplaincy in prisons in three German federal states (*Länder)*. Balancing the normative requirements of both religious and public institutions (Sullivan, 2014), prison chaplaincies provide ‘a lens through which to explore critical questions relating to contemporary religion in public life’ (Gilliat-Ray, Ali, & Pattison, 2013). Germany constitutes a particularly interesting case for the research question at hand, since its *Länder* dispose of substantial autonomy in regulating religious diversity while sharing a similar constitutional framework. Allowing one to observe varying intra-national mechanisms while holding the institutional context constant, subnational comparison highlights the interplay between national legal frameworks, institutional settings, and subnational political dynamics. Examining the formalisation of Muslim prison chaplaincy in Lower Saxony, Berlin, and Hessen, I have focused on those subnational cases where this process appears most advanced to date. Formal establishment of Muslim chaplaincy in Germany seems particularly surprising, as, until recently, state actors argued that a formalised, area-wide service of Muslim care in prisons could not be provided given that Muslim communities had not yet fulfilled the mandatory legal requirements of the German state-church law. How then can we explain the emergence of an organised Muslim chaplaincy in the three aforementioned *Länder*? Moreover, how do national legal frameworks intersect with institutional practices and subnational configurations in the construction of Muslim chaplaincy?

My argument proceeds in two analytical steps. It first traces back how various religious and state actors have drawn on regionally diverging strategies of public reasoning (Bowen, 2010, p. 3) regarding Muslim chaplaincy in order to accommodate the legal provisions of state-church relations. It thereby compares varying framing patterns for the right to Muslim chaplaincy. Building on this first level of analysis, the article, in a second step, moves closer to the concrete institutional setting of the prison. It asks how framing strategies constructed in order to respond to national legal challenges might ultimately also affect the chaplain’s position *within* prison walls – especially in situations of institutional tension. The chaplain is indeed one of the rare actors in prison who dispose of extensive rights without formally being part of the prison administration. Acting in a religious mission and partly escaping the security imperative of the prison, the chaplain balances the requirements of this institution on the one hand and the freedom of religion on the other. The chaplain’s right to refuse to testify constitutes a central status element expressing this tension. I have demonstrated to what extent the ‘legal uncertainty’ inherent to this right opens up a space for renegotiating the institutional realm wherein the chaplain has to find his place. As the comparison between the three *Länder* demonstrates, this space is shaped differently depending on the public reasoning that initially lead to the formalisation of prison chaplaincy.

The paper is structured as follows: First, I have provided an overview of existing research on religious diversity in prisons and have advanced my own theoretical and methodological argument (1). The paper then gives a short description of the German legal context and the current developments regarding Muslim chaplaincy and methodology of the present research (2). The remainder of the article exposes the analysis of the empirical material in two steps: first, the accommodation of national state-church patterns through varying framing strategies regarding Muslim chaplaincy (3) and, second, the negotiation of the chaplain’s role at the intersection of the security dimension of the prison and the religious freedom he enjoys (4). A concluding discussion addresses the insights the study of the governance of religious diversity might gain from subnational comparison (5).

## (1) Conceptualising the institutionalisation of Muslim prison chaplaincy through subnational comparison

### Religion in prison

Diversification of Europe’s religious landscape does not stop at the prison door. Since the path-breaking study Beckford and Gilliat conducted about the accommodation of religious diversity in English and Welsh prisons (Beckford & Gilliat, 1998), an increasing amount of literature has addressed religious diversification in the prison context. Authors have extensively examined specificities in the practices and perceptions of religion among inmates and prison agents (Becci & Knobel, 2013; Béraud, Galembert, & Rostaing, 2016; Lamine & Sarg, 2011), profiles of Muslim chaplains (Beckford, Khosrokhavar, & Joly, 2005; Rhazzali, 2015; Schneuwly Purdie, 2011), and the multiple material and non-material locations of religion in prison (Becci, 2011). Of immediate and central interest for this work, many authors have assessed the political, legal, and prison-intern processes leading to the emergence of Muslim chaplaincy in different national contexts. The literature reveals at least four dimensions that are central to the development of Muslim care in prison. (1) The strong imprint Christian heritage has left on newcomer religions in prison has been throughly documented by now. Thus, not only do Christian chaplains often serve as central bridges for Muslim caregivers to access the prison (Beckford & Gilliat, 1998; Furseth, 2003), but the chaplaincy model, as profoundly entangled with the Christian community’s structure and thinking, also influences the ‘social forms’ of minority chaplaincies (Beckford, 2015, p. 22). However, researchers have also found dynamics of mutual influence to emphasise the overall restructuring of chaplaincy models through religious diversification (Ajouaou & Bernts, 2015; Beckford & Gilliat, 1998). (2) Furthermore, the absence of clearly established structure and leadership in Muslim communities in Europe has been highlighted by various authors as a major challenge for the establishment of chaplaincy service in prison. The introduction of Muslim care in prisons therefore seems to go hand in hand with a restructuring of the community itself (Al Asri, 2015).

(3) A very prominent claim within the research on prison chaplaincy concerns the imprint left by the national specificities of regulating religious claims. The chaplaincy constitution has been considered as a microcosm reflecting national legal and political contexts (Roy, 2015), and many studies have held that national norms of state-church relations directly influence when, how, and why Muslim chaplaincy emerges (Becci, 2011; Beckford et al., 2005; Rostaing, Béraud, & Galembert, 2015). However, researchers have also pointed to the limits of this influence. Some have challenged the assumption of ‘path dependence’ by suggesting that a discrepancy between governance of religion and Muslim prison chaplaincy might appear due to political tendencies, such as the ‘perceived urgency of the radicalisation of Islam’ (Furseth & Kühle, 2011). In this sense, Furseth and Kühle have argued that ‘there is no necessary direct link between overall policies on multi-religious societies and the actual accommodation of Muslims and other religious minorities in public institutions like prisons’ (Furseth & Kühle, 2011, p. 121). Others have highlighted that a pragmatic view of religion as a vector of security can – on a case-by-case basis – lead prison agents to grant space for religious practice (Rostaing et al., 2015). This points to the fourth dimension present in the literature.

(4) Analysis of the imprint left by prisons as ‘total institutions’ (Goffman, 1961) on the mission of the chaplain occupy indeed a particularly prominent place in the existing literature on prison and religion (Becci, 2015). Authors have examined the multiplicity of functions a chaplain can assume within this total institution, as well as the limitations that the omnipresent security imperative imposes on him (Becci, 2014; Rostaing et al., 2015). The chaplain’s position appears to be one that balances permanent institutional tension: Thus, while the prison as a ‘total institution’ (Goffman, 1961) and an apparatus transforming the individual (Foucault, 1993) follows a logic of total surveillance and security, religion and its institutionalisation via the right to freedom of religion limits this totalitarian ambition. Prisons therefore face the paradoxical challenge of exercising integral control of interactions while simultaneously having the obligation to ensure the right to free exercise of religion (Becci & Knobel, 2013). The (Christian) chaplain is the direct expression of this tension. While his extensive rights (access to the cells, confidentiality of exchanges, possession of keys, right to secrecy) and his independence from the prison administration (Eick-Wildgans, 1993) allow him to partially escape the security restriction of the prison (Béraud, Galembert, & Rostaing, 2013), Muslim chaplains still have to negotiate their position in the institution. Individual attitudes of prison agents and the locally varying bridge-function of Christian chaplains seem to play a crucial role in this respect (Beckford et al., 2005; Béraud et al., 2016).

In sum, researchers have provided very rich insights into emerging chaplaincy profiles in multi-faith environments. However, while most of these works mention and illustrate several of the four contextual dimensions mentioned above, their interaction and possible strategic mobilisation by actors negotiating and framing the inclusion of minority religions has not yet been systematically assessed. Indeed, how are prison-specific logics and national ideologies articulated together in the formalisation of prison chaplaincy? And to what extent does this articulation vary according to regional actor constellations?

In the German context, Sarah Jahn’s work illustrates some of these challenges. Examining an evangelical organisation and Jehovah’s Witnesses, Jahn has found that national legal requirements regarding state-church relations matter less for the integration of religious newcomer communities in the prison than the prison-specific logic of rehabilitation itself. Decisive for integrating religious groups into the prison structure, she has argued, is their perception as a factor of rehabilitation. In several publications, Jahn has mentioned the ongoing institutionalisation of Muslim prison chaplaincy (Jahn, 2013, 2014, 2015). However, it is not yet clear how exactly this institutionalisation happens, whether they are also – similar to the examined evangelical group – perceived as rehabilitation actors, and through which mechanisms Muslim actors overcome legal and political challenges. In particular, the question of German federalism has not yet entered analytical considerations.

In this paper, I therefore have sought to connect the research on religion and prison to a recent theoretical trend that has conceived of the study of governance of religious diversity as a dynamic interplay of various institutional, political, and legal logics. Displaying this approach through subnational comparison sheds light on actor strategies shaping this interaction.

### Governance of religious diversity from a subnational perspective

Moving beyond earlier accounts of the governance of religious diversity that emphasised national models (Fetzer & Soper, 2004) or larger transnational frameworks (Koenig, 2007), more recent theoretical work on said governance has been paying closer attention to the specific role of institutional settings. Institutions such as the military, hospitals, courts, and schools have been conceived of as governed by the location-specific rules, practices, and boundaries that influence how religious diversity is regulated (Bowen et al., 2014). Bowen and his colleagues have thus accentuated ‘the dual character, national and institutional, of the mechanisms and processes shaping perceptions and boundaries regarding Muslims in Europe’ (Bowen et al., 2014). The authors have relied on the sociology of institutions, which demonstrates that institutions, understood in a broad sense as both rules and cognitive patterns, and concrete organisational settings, do not exist as static blocs but are internally negotiated by the actors that inhabit or make use of them.^1^ Drawing on these insights, the authors have asked to what extent national ideologies – including patterns of state-church relations – and the specific organisational norms of concrete institutional locations are mutually shaped in the process of diversity incorporation.

While building upon this approach and extending it to the prison context, which is surprisingly absent from the cross-institutional comparison by Bowen and his colleagues, this article suggests a more fine-grained analysis, by bringing into the play the role of subnational contexts characterized by varying actor configurations.

Subnational comparison constitutes a dimension that has not yet been extensively explored by social scientists interested in religion. Helbling and Traunmüller recently published a study assessing attitudes towards immigrants in Switzerland depending on the state-church frameworks varying from one canton to another (Helbling & Traunmüller, 2015). This study advocates subnational comparison in Switzerland as a tool for improving cross-national comparison by examining the impact of diverging legal settings within a constant political and economic context. I suggest using subnational comparison in cases displaying the reverse logic, e.g. in cases that are characterised by strong federal structures but similar legal settings in regional units. Germany constitutes an exemplary case in this respect. Indeed, whereas cross-national comparison runs the risk of supposing national heterogeneity where there is not (Snyder, 2001), focussing on subnational comparison here allows to take into account regional variations regarding governance and accommodation of intra-nation tensions while holding the state-church context constant. Subnational comparison seems particularly useful when considering that institutional locations and national legal settings do not constitute ‘coherent’ blocs, but are challenged by local and transnational dynamics and contain intrinsic tensions as well (Bowen, 2007). Emphasising the active and strategic role of state agents and Muslim entrepreneurs, the comparison of different local sites might bring these processes of accommodating inherent tensions in different ways into sharper focus for both levels of analysis pursued in this article: the dynamic interaction with national legal frameworks on the one hand and the institutional location of the prison on the other.

As regards the overcoming of national legal barriers in the formalisation process of Muslim care in German prisons, one might think of at least two framing strategies that actors could possibly draw on in public struggles for recognition. First, by mobilising the prison’s priority of security and rehabilitation, the perception of religious organisations as ‘rehabilitation actors’ could, if we follow Jahn’s suggestion, ‘overrule’ the legal criteria of corporatist church law. Second, the rising institutional power of individual rights discourse and its increasing penetration of prisons (Galembert & Rostaing, 2014) also constitutes new opportunities upon which Muslims acting at the grassroots level could draw in order to accommodate corporatist church laws from below. Framing strategies might vary depending on the concrete local situation – in terms of networks, power, and resources – of Muslim actors. Thus, while well-established and organised actors might be able to afford to claim equal status as compared to Christian communities, less well organized and connected actors might tend to draw on alternative framings. Shedding light on their variation within nationally common legal contexts, subnational comparison brings us closer to these actor strategies and constellations.

Furthermore, it may be safely assumed that, depending on the framing mobilized by actors in order to create an access for Muslim chaplains to the prison, their position *within* the ‘total institution’ is differently shaped when facing its intrinsically conflicting normative requirements between freedom of religion and the security imperative of the prison. While a socio-political ‘rehabilitation’ framing might ‘disintegrate’ the Muslim chaplain from the religious realm by assimilating his mission to the aims of the prison itself, an equality- or (individual-) rights-based framing might tend to reproduce the freedom and independence the Christian chaplain would traditionally enjoy. These processes should become particularly visible in situations of ‘uncertainty’ regarding the rules that define the margins of action within the prison. The reviewed literature on chaplaincy has indeed shown that the incorporation of religious diversity bears the question of how to apply a legal construct – shaped on the ‘model’ of cooperation with the Christian churches - to religious newcomers. This ‘transfer’ possibly opens up spaces for renegotiation of statutes and liberties. Subnational comparison demonstrates whether and to what extent these negotiation processes vary along the regional lines corresponding with the framing strategies of different actor constellations.

## (2) Muslim prison chaplaincy in Germany: contextual background and methodological approach

Before turning to the analysis of my empirical findings, a brief overview of the German state-church framework and the formalisation of Muslim chaplaincy seems necessary. Until recently, Muslim chaplaincy has been a local and rather random phenomenon in German prisons. Muslim volunteers intervene occasionally or are recruited on a local basis (Jahn, 2013). Various Muslim organisations from the quite diverse Islamic landscape in Germany (Rosenow-Williams, 2012) have sometimes established more regular local modes of cooperation with individual prisons. Despite these ‘interim solutions, […] there is still no general *modus vivendi* based on the law and administration principles’ (Jahn, 2015).

The requirements of German corporatist church law appear to be the most important barrier to the institutionalisation of Muslim chaplaincy (Jahn, 2013). Building on the legal recognition of religious communities (*Religionsgemeinschaften*) that fulfil specific criteria of representation and organisation, the corporatist church law has established a cooperative model between religious communities and the state. The question of being recognised is a matter of the individual state (*Land*). Once a religious group has received legal status, the cooperation between the *Land* and the community is organised on the basis of contractual agreements. Within this framework, recognised *Religionsgemeinschaften* are allowed to conduct religious classes in public schools, run cemeteries, participate in the national media, and establish chaplaincy services in public institutions (Classen, 2003).^2^ While the *Länder* follow very similar formal requirements of legal recognition, the precise modalities of cooperation are regulated autonomously and therefore potentially vary from one *Land* to another.

Even though legal scholars have already discussed the possible openings of the constitutional framework for the integration of Muslim organisations (Hennig, 2010; Joppke & Torpey, 2013), the requirements mentioned above still constitute deeply-rooted administrative ‘traditions’ that dominate practices and thinking in the public sphere. The Ministry of Justice in Hessen, for example, joins previous federal government statements,^3^ by underlining that the difficulty of establishing area-wide Muslim chaplaincy is due to the absence of any unified Muslim interlocutor.^4^

Nevertheless, since 2010, formal negotiations aimed at institutionalising prison chaplaincy service have been initiated in several *Länder*. In the following, I have focused on those three *Länder* where this process has been pursued most extensively. In Lower Saxony, a contract regarding the creation and the modalities for the realisation of a Muslim prison chaplaincy was negotiated in 2012^5^ between representatives of the Ministry of Justice and of the two main Muslim organisations there, the DITIB (Turkish-Islamic Union for Religious Affairs) and SCHURA.^6^ In Berlin, broad contractual negotiations and chaplaincy training with 30 Muslim volunteers were conducted between 2011 and 2014. The contract that was elaborated in this context has finally not been enacted. However, after three years of preparation, a report from the Federal Office for the Protection of the Constitution (*Bundesamt für Verfassungsschutz*) about those 30 individuals trained for prison led to the interruption of the project, leaving the contract unsigned.^7^ In the following, I have not explored the implications of this failure, but have focused on negotiations leading to the project initialisation and conception.

Even though formal negotiations have been envisioned by the ministry of Justice in Hessen at the regional level, they have not yet reached an advanced stage. However, two local modes of cooperation have been established in the prisons of Wiesbaden and Frankfurt. The first is of particular interest here, because it has been referred to by the Ministry of Hessen when questioned about an area-wide institutionalisation.^8^

The present study has mainly built on qualitative, in-depth interviews conducted between January and March 2014 with those actors who had been involved in the different initiatives.^9^ In Lower Saxony, I met state officials at the Ministry of Justice as well as official representatives of Muslim organisations who had negotiated the contract about the establishment of an area-wide Muslim prison chaplaincy. In addition, I interviewed Christian chaplains and imams who have already been working as volunteers in prisons in Hanover. In Berlin, interviews were conducted with an even broader range of actors, as the project involved prison officials, Ministry of Justice officials, the Expert Council for Integration and Migration of the Berlin Senate (*Beauftragter des Senats von Berlin für Integration und Migration*), nine Muslim organisations, experts on the prevention of violence, and a civil society organisation. In Wiesbaden, the Muslim chaplaincy project involved a highly active ‘coalition’ of three entrepreneurs: a locally intensively engaged imam, the director of the prison in Wiesbaden, and the council of integration of the city. All three actors were interviewed in the framework of the present study. Interviews have furthermore been conducted with a legal expert advising Muslim communities in Germany and Christian and Muslim chaplains engaged in the public debate about Muslim chaplaincy. Documents and articles published by some of the interviewees have constituted additional empirical material. The interviews and documents have allowed me to trace back the public reasoning regarding the institutionalisation of Muslim prison chaplaincy.

## (3) Group equality, individual rights, and prevention of radicalisation – Three pathways to Muslim prison chaplaincy

Why and how has the formalisation of Muslim prison chaplaincy become possible? Through what strategic public reasoning have legal obstacles to the institutionalisation of Muslim prison chaplaincy been overcome in the three *Länder*? The comparison highlights that actors in different regional contexts substantially diverge in how they frame Muslim prison chaplaincy. I was indeed able to observe three very different patterns within the same legal context: In Lower Saxony, Muslim prison chaplaincy emerged as part of a broader struggle for rights equality. Path-dependent, top-down developments regarding the institutionalisation of Islam has led to the constitution of Muslim chaplaincy as one milestone of this overall aim. The initiatives in Hessen and Berlin, in contrast, have followed a bottom-up dynamic wherein legal challenges have been respectively accommodated through a socio-political framing of chaplaincy related to the needs of the prison structure and the emphasis of an individual right to religious care.

### Lower Saxony

In Lower Saxony, the establishment of Muslim chaplaincy resulted from a top-down institutionalisation process of Islam. This process has followed the pre-traced state-church pattern. Indeed, when asked to recall how the negotiation of the contract was initiated, both of the organisations involved in the process (SCHURA and DITIB), as well as representatives from the ministry, unanimously pointed to the particular political opening created by the former prime minister Christian Wulff in 2005, after the celebration of the 50^th^ anniversary of the contract between the churches and the federal state of Lower Saxony.^10^ Wulff’s suggestion to initiate a similar relationship with Muslim organisations through formal cooperation fell on fertile ground, given that the Muslim community was, at the time, actively pursuing the establishment of internal structures corresponding to the requirements of corporatist church law. They indeed set up local structures unifying around 90% of the mosques under two umbrella organisations (SCHURA and DITIB). 11 Established relations between the churches and the state directly inspired the claims brought before the regional government. A report from one of the main initiators of the institutionalisation process is elucidating in this respect:

[T]he ‘Grüne’ [Green party] suggested, in North Rhine-Westphalia, clarifying the debate about the tentativeness of Muslim organisation to create federal structures […]. In this context, Professor Heinrich de Wall has written a report regarding the important points that have to be considered in a contractual relationship between Muslim organisations and the region of North Rhine-Westphalia (De Wall, 2004) – in analogy with the already existing Church contracts. […] This paper and the ideas of Wulff allow us to think about what we – SCHURA Lower Saxony – would have to do in order to obtain such a contract.

[W]e had the first meeting at the ministry, and the question was what we would like to integrate into such a contract. And who was the only person prepared? Me. Sorry to say it like that, […] but, as we [SCHURA Lower Saxony] had already discussed a bit, I brought a paper with the aspects we should regulate […] A list with 27 points.^12^

Figuring among these 27 points, Muslim prison chaplaincy very soon became one of the negotiation chapters – next to religious classes in public schools, for example – set up on the path to a state contract between the *Land* and SCHURA and DITIB. In this line of reasoning, both interlocutors suggested that the central motivation for formalising Muslim care in prison is grounded on a more general claim for equal rights. This legal equality is essentially thought to be achieved by reproducing the organisational paths of the churches. In this sense, the president of SCHURA underscored: ‘We had a list of points to negotiate. We looked at what other religious communities (*Religionsgemeinschaften)* had and what we did not have.’^13^ Similarly, the lawyer representing the ministry in the negotiation process also framed the newcomer chaplaincy in terms of legal equality among different religious groups. He stated:

I would not have conducted the negotiations differently with the Catholic Church. And I would not have conducted it differently with the Jewish community. I think it was helpful that I did not try to interfere with the contents of the different *Religionsgemeinschaften* or to leave the expert knowledge up to them, but to apply the same legal frame to each of them and not to differentiate where it was not necessary.

Later, he added:

And I would be very happy if in 5 or 10 years we look back and we can say that we reached the same situation as with the Catholic and Protestant churches.

This framing in terms of group equality has been present in multiple other aspects of the contractual negotiations. The prison administrations, for example, explicitly distanced themselves from any political considerations regarding religious extremism in their hiring procedures for the chaplaincy by referring to the equal neutrality of the state towards all religious groups. The group equality framing also became manifest in the choice of appellation for the new religious care. The use of the term of ‘chaplaincy’ was controversial and debated, particularly with respect to its Christian heritage and privileges. However, state actors insisted on state neutrality regarding the regulation of theological content and concepts and therefore left it up to the Muslim community to choose their appellation.

### Berlin

The case of Berlin constitutes a striking contrast compared to the developments in Lower Saxony. Rather than legally framed top-down group ‘equalisation’, one can observe a bottom-up process with an individual rights claim emerging from the prison itself and leading to a project-centred approach. The claim of getting access to an imam advanced by individual prisoners in Berlin has been channelled via representatives of detainees to the Expert Council on Integration and Migration and to the media.^14^ An organisation involving diverse state and civil society actors that regularly debate questions concerning prisoners with a migrant background (*freiabonnements* e.V.) subsequently decided to make Muslim religious care in Berlin prisons one of the topics debated at its regular ‘roundtable’. From this initiative arose formal negotiations regarding a contractual and publicly funded institutionalisation of Muslim prison chaplaincy, connecting diverse actors, such as prison directors, seven Muslim organisations (partly specialised in chaplaincy)^15^ that compose the main local Muslim landscape in Berlin, civil society actors with expertise in the domain of violence prevention and social work, and politicians, as well as church representatives. This actor constellation points to the prison-need-centred design of the initiative. Negotiations have indeed been conducted without considering the absence of a legally recognised religious partner (*Religionsgemeinschaft*) as an obstacle. In this respect, a legal expert regularly consulted by Muslim communities in Germany underlined:

Valuating the constitutional right to freedom of religion […] rather than formal constitutional requirements, project cooperation might be a step towards progressive development of formal relations in other domains.^16^

Illustratively, the official interlocutor in the Ministry of Justice in Berlin referred to the penal code, ‘which simply states that the prisoners have to be supported in the exercise of their religion’. In his view, the code certainly expressed an individual right, but the exact form of realisation remained open.^17^ Privileging individual rights in prison by setting up cooperation involving manifold actors confronted in their everyday work life with prison needs, practices, and rules, the initiative took a pragmatic shape. Indeed, the framing of prison chaplaincy as an ‘apolitical’ project centred on the precise needs of the prison has also been a condition for reassembling the Muslim interlocutors: ‘Chaplaincy is something with which all of us can identify. We are all concerned, and it is not something political. It concerns the individual, the human. Therefore, we are willing to work together.’^18^

Muslim organisations in Berlin did not – as their counterparts in Lower Saxony – consider chaplaincy as a milestone of a general agenda of rights equality, but, foremost, as an individual religious need. While this pragmatism opened ways of accommodating Muslim rights, addressing legal requirements by setting up a pragmatic pilot project however also created some space for maintaining the difference between Muslim and Christian chaplaincy explicitly denied in Lower Saxony. Thus, Muslim interlocutors accepted the government officials’ insistence on institutionalising Muslim chaplaincy under the appellation of ‘religious care’, leaving the notion of ‘chaplaincy’ exclusively to their Christian counterparts. The administration argued:

The notion of ‘chaplaincy’ belongs to Christian chaplains, but also to psychologists. With both, one associates a concept and also a specific formation. […] So, the imams, which means those who would be qualified to take over the tasks of chaplaincy, if one takes the viewpoint of a Christian chaplain, they do not really have the necessary profile […]. An imam can be a chaplain, but it depends on the person. […] A concept still has to be developed.^19^

The Muslim side pragmatically renounced to the appellation of ‘chaplaincy’:

[T]his was the first time that this debate emerged, when we met representatives from the Christian churches. They said it was a Christian term and so on. First, we were really surprised. […] At first, we did not really understand why we were discussing this term. But we understood that it was something political, and that there are rights that are coupled with Christian status. We did not understand […] You take care of somebody’s soul. Everybody has a soul […]. But I don’t care, really absolutely not. I am pragmatic. We just want to help and not define everything.^20^

This debate exemplifies that the legal inclusion reached through the pragmatic shape of the initiative is symbolically ‘counterbalanced’ by a distancing of the roles of the Christian and the Muslim chaplains. This distancing ultimately becomes possible by conceiving of chaplaincy as a form of cooperation limited to a pilot project that, as such, was not part of a broader recognition process.

The cooperation project of Berlin has been interrupted by the Senate of Justice at the end of the year 2013 after a phase of long preparation and negotiation. The interruption followed a report established by the German *Verfassungsschutz* regarding the about to be Muslim chaplains considering that central actors of the project presented security risks. Whether the until then negotiated modalities will influence the recent tentative to initiate a regular Muslim care in Berlin prisons^21^ however goes beyond the research of the present article.

### Hessen

The case of Wiesbaden in Hessen neither follows the path-dependent model observed in Lower Saxony nor the individual rights scheme prevailing in Berlin. Rather, Muslim chaplaincy is framed as a means for rehabilitation and, in particular, as a struggle against religious extremism in prison. As such, it reflects both the profile of the individual entrepreneurs involved and the strategic circumvention of state-church requirements. The local developments have evolved mainly around three pillars: the prison director, a locally engaged imam and the Council of Integration. First, the personal engagement of the prison director has been an essential motor for the  initiative. Driven by the ambition to fulfil the right of each prisoner to access religious services, but strongly influenced at the same time by a previous negative experience with a radicalised imam in another prison, she contacted the Council of Integration of the City of Wiesbaden in order to find an imam for the prison. Second, the city of Wiesbaden itself wanted to set an ‘example’ for integration through a contract (*Integrationsvereinbarung*) with local Muslim organisations in order to promote integration on the basis of a set of ‘shared values’.^22^ Third, the central Muslim entrepreneur in this project, the convert Husamuddin Meyer, has been strongly committed to the prevention of Salafism, in light of which he reads his religious mission in prison.^23^ The shared political framing of religious work led the three local actors – that is, the prison director, the city of Wiesbaden, and Husamuddin Meyer – to initiate the formal institutionalisation of Muslim prison chaplaincy in Wiesbaden and to obtain a more substantial budget allocated by the Ministry. Not surprisingly, the proposition made by this ‘local coalition’ to the regional Ministry of Justice and Integration advocates a view of chaplaincy strikingly different to the cases of Lower Saxony^24^ and Berlin.

The situation of lots of young men with an immigration background is characterised by difficulties at school, conflicts between the expectations of parents inspired by their cultures of origin and the expectations of our society. In addition, the economic situation of their parents’ household is often very difficult. The criminality of lots of these young men with an immigration background results from a failure of the integration process. Furthermore, the experiences show that their religious knowledge is often rudimentary and ‘erroneous’. This is an obstacle for integration. […] Spiritual Muslim care could help in two regards: prevention and promotion of integration.^25^

This proposition not only frames Muslim prison chaplaincy as a contribution to social integration and rehabilitation, but also serves as a strategy in order to ‘circumvent’ the requirements of corporatist church law which could not be met by individually organized actors. In this respect, the statement from the representative of the Council of Integration is illuminating:

There have been different attempts at formalisation [of Muslim chaplaincy]. On the one hand, we tried to introduce it through an ‘intercultural opening’ of the prison – that’s what we called it. We even tried to conceive a global project including all the intercultural dimensions of the work in the prison. […] To be honest, it was also a kind of means to an end. Each time we failed because of the argument that the churches had contracts and played a particular role in Germany, that there are structures that have evolved over centuries, and that there is another judicial basis in these cases. […] There are attempts to establish equal structures by Muslim organisations, but I think it will still take a lot of time. […] And this is why we thought that by introducing a general ‘intercultural opening’ of the prison, the subject of Muslim chaplaincy would have been less in the spotlight. But it didn’t work out.^26^

Advocated by the ‘local coalition’ and especially by Husamuddin Meyer,^27^ the socio-political approach to chaplaincy as a facilitator of integration and rehabilitation also seems to have inspired the initial steps of regional reflection on the institutionalisation process. The Ministry conceives of the latter as a combination of religious ceremonies and the social work carried out by chaplains ‘representing the constitutional values of our society’.^28^

The socio-political framing led the prison director to consider Muslim chaplaincy as different to Christian chaplaincy and to prefer, similarly to her counterparts in Berlin, the notion of ‘religious care’ to the term ‘chaplaincy’. She noted: ‘The Christian churches have chaplains, and the Muslim chaplain carries out religious assistance. They have the same rights, but it might be very different regarding the tasks to be accomplished.’^29^

In sum, the subnational comparison of the three cases has brought to light a clear variation regarding the construction of the right to Muslim chaplaincy within the same national legal setting. Three patterns of public reasoning vis-à-vis the constitution of a Muslim chaplaincy have become visible: path-dependence regarding state-church relations (Fetzer & Soper, 2004) putting in advance an equalisation among different religious groups, pragmatic accommodation of the legal framework through individual rights claims linked to practical ‘need’ in prison, and the legal framework’s circumvention through ‘politisation’ of the function of the chaplain as part of the rehabilitation mission of the prison. The last two cases illustrate, in two distinctive manners, Bowen and his colleagues’ assumption that specific institutional settings influence the reading and possible accommodation of national ideologies, whereas the first case rather fits an understanding of religious governance as strongly affected and guided by national ideologies of state-church relations.

## (4) Between freedom of religion and security imperative: Negotiating institutional tension

How do the different framings of the right to Muslim chaplaincy influence the position of Muslim chaplains *inside* the prison? More precisely: How do they play out in situations of institutional tension? In this section, I have moved closer to the prison location by analysing one important component of Muslim prison status. I have argued that the overall framing of Muslim prison chaplaincy plays out differently in situations of institutional tension and uncertainty.

As outlined above, chaplains are anchored in two potentially conflicting realms: the freedom of religion on the one hand and the prison institution seeking to restrict any possible freedom on the other. Even though many of the specific rights a (Christian) chaplain enjoys are rooted in traditions rather than in legal rules, there is at least one crucial, legally binding element that defines the chaplain’s status: the obligation of secrecy and the right to refuse to testify (*Zeugnisverweigerungsrecht*). As has been noted by a Christian chaplain, ‘The prisoner would not place that much confidence in our work if we did not have this right. […] It is also something that makes us incalculable for the prison administration.’^30^

Interestingly enough, although the right to refuse to testify is anchored in the penal code and is not part of the specific contractual relationship between religious communities and the German state, it has been discussed in all three chaplaincy initiatives. Constituting the legal expression of the balance between the centrifugal forces of freedom of religion and the security dimension intrinsic to prison, the right to refuse to testify faces permanent tension. This tension is doubled by ‘legal uncertainty’, regarding the question whether – legally - other religious actors than Christian clergymen can access this right. A debate that took place in 2008 at the national level demonstrates this ‘legal uncertainty’ inherent to the right to secrecy. In the general political climate of ‘securitisation’ (Eckert, 2008) surrounding Islam since 2001, politicians have brought to the forefront a public policy proposition integrating anti-terrorism measures in the Federal Criminal Police Office law (*Bundeskriminalamtsgesetz*). While the law explicitly excludes – under the protection of the right to refuse to testify – ‘clergymen’ from secret hearings and other measures of surveillance, the question of whether imams should or would be protected under this same statute has triggered much political controversy.^31^ The debate, having not yet been definitely clarified, embeds the prison chaplaincy question in a nationally discussed policy regarding anti-terrorism. But the question of granting the right to refuse to testify to those of religious denominations other than Christians has not only been discussed in the legislative sphere but has also been brought to the courts. Thus, in a dispute pertaining to individuals from the Yezidi minority, the Constitutional Court decided that the Yezidi religious officials involved in the dispute were acting as ‘clergymen’ and therefore could ultimately rely on the right to refuse to testify.^32^

These legal and judicial debates point to the margins and fragile balance inherent to the right in question. Comparing the different Muslim chaplaincy initiatives, the ‘legal zone of uncertainty’ inhabiting the right to refuse to testify has been occupied very differently depending on the cognitive framings applied to Muslim chaplaincy institutionalisation. Tracing back the discussions regarding the practical modalities of chaplaincy in all three contexts reveals to what extent it has contributed to a renegotiation of the balance between totalitarian security and freedom of religion when it comes to the integration of religious diversity in the institutional setting of the prison.

### Lower Saxony

The representatives of DITIB and SCHURA very much insisted on including a clause regarding the right to refuse to testify in the negotiated contract. Considering this right to refuse as essential for legal equality achievement, they emphasised: ‘[T]here cannot be two rights systems in one country. And there cannot be a double-tracked jurisdiction. Therefore, we insisted on this right; otherwise, we would not have participated [in the contract negotiation].’^33^ Yet, the ministry was reluctant, given that no individual state contract can substitute or create national law. In its view, only courts have the official power to decide on this question on a case-by-case basis. Therefore, the final agreement of introducing a clause stipulating that the right in question was assured if the Muslim chaplain acted as a clergymen, as well as the reference to the Yezidi case,^34^ were considered by the legal department of the Ministry as a symbolic agreement that would be potentially helpful for the Muslim organisations regarding the broader policy implications pointed out above. In this sense, the interlocutor in charge of the contract elaboration mentioned that the clause ‘was thought to be a concession to the Muslim organisations and maybe useful for them in order to advance in the national debate’.^35^ Muslim actors indeed have explicitly emphasised their hope to promote their rights at the national level through the negotiations at the local level. Once again, Muslim chaplaincy appears to be a strategic step on the way to broader equality among Christian and Muslim communities. In this case, the framing of the chaplaincy initiative in terms of legal group equality has led to the reproduction of the freedom of Christian chaplains, meaning that the institutional balance between freedom of religion and security has been maintained for Muslim chaplains. The inclusion of the right to refuse to testify in the contract – even if only symbolic – has opened the door for Muslim prison chaplains to experience an autonomy similar to their Christian counterparts.

### Berlin

In Berlin, the negotiated but finally not validated contract introduced a very different clause. It explicitly stated that imams and spiritual caregivers should communicate information to the prison administration if it might protect the life of a person, prevent real danger to the health of a person, or is of interest for preventing significant criminal acts.^36^ The prison administrations opposed the guarantee of the right to refuse to testify and insisted on introducing a clause contrary to the negotiated contract. Thus, the prison administration underlined:

The question of the right to secrecy has been heavily debated. Because they [Muslim chaplains] are not clergymen who have this right and they are not doctors neither. […] Finally, just a very small circle of people has this right. Doctors and also our Christian chaplains – I say it like this now – have the right to refuse to testify. But we really do not want to enlarge this group. Because there are always security incidents and we depend on each person who can help us.^37^

This argumentation not only reflects the symbolic distance that was part of the overall framing of Muslim chaplaincy, considering Muslim ‘assistants’ as not yet equal to Christian chaplains, but also demonstrates that the administration has drawn on this distancing in order to give greater prevalence to the security mission of the prison. Even though the religious dimension of Muslim spiritual care has not been denied, the institutional balance seems to have shifted towards a greater emphasis on the security (and rehabilitation) dimension of the chaplain’s function.

While some of the Muslim actors trained to become a chaplain are not even conscious about the existence of this right, those who have been directly involved in the contractual negotiations pragmatically have accepted potential restrictions on this level. In this sense, the main Muslim representative of the initiative mentioned that ‘[they] just wanted to obtain the contract and later [they] could try to negotiate more rights. […] These are procedures that cannot be changed easily and [they] understand perfectly.’^38^

Paradoxically, while the pragmatism consisting in a flexible reading of state-church requirements by relying on individual religious rights in prisons gave greater emphasis to the practical needs in prison and the right to freedom of religion than to formal constitutional requirements, it also opened up a margin of negotiation for differentiation between established religious groups and religious newcomers inside prison walls based on cultural and political considerations.

### Local practices

As no formal contract has been negotiated in Hessen up to now, the question of the right to secrecy has not emerged as an explicit issue of the chaplain’s role. However, the case invites one to turn to the question of to what extent this provision plays a role in concrete practices within prisons. Indeed, situations where the right to refuse to testify is mobilised appear to be extremely rare. Moreover, a number of Muslim individuals involved in the different local context were even not conscious about the existence of this right.^39^ However, practices in Berlin prisons as well as in Wiesbaden (Hessen) have shown that the right to refuse to testify is perceived in light of a symbolic distance to Christian chaplaincy. In this respect, chaplains in Berlin and in Hessen underscored:

The question of the right to refuse to testify has not yet been resolved for Muslim chaplains. We think that we cannot be sure that the right is valid for us. […] Pastors clearly have this right in any case, but we do not […]^40^

We do not yet have the right to secrecy. However, I do not talk with the prisoners about their secrets. If there were a real problem, I would talk about it with the director, if it were something really dangerous and severe. But normally, I don’t.^41^

All actors working for the prison administration emphasised that they normally respected the right to secrecy of all religious workers. The question of the right to refuse to testify becomes important only in extreme cases, such as those indicated in the negotiations in Berlin (cf. above). Additionally, even Christian chaplains have to reconsider their obligation to secrecy when it comes to severe problems regarding the safety of prisoners.^42^ However, the uncertainty maintained in the case of Muslim chaplains broadens the legal margins and challenges the balance between religious freedom and surveillance. In the meantime, framings of Islam and its institutionalised practises – be it an overall equality frame, an individual need and rights frame, or a rehabilitation frame – potentially do play an important role in how this space of legal uncertainty is occupied in a situation of institutional tension.

## (5) Discussion and Conclusion

The aim of this article has been to examine the dynamics of the governance of religious diversity as an interplay of national and institution-specific sets of logic by looking at the construction of Muslim prison chaplaincy in Germany. The subnational comparison has focused on framing strategies that have allowed Muslim communities to overcome legal boundaries anchored in the German corporatist state-church model and the impacts of these framings on the institutional modalities of chaplaincy practice within prison. Even though observing the initial moment of emergence of Muslim chaplaincy does not allow to assess routines and stabilized modes of interaction, it sheds light on dynamics and mechanisms which articulate the negotiation of the place of religious newcomers at the intersection of law, institutional frames and specific actor constellations.

In two of the three cases (Berlin & Hessen), the emergence of chaplaincy from the practical context of the prison has led to a flexible, bottom-up accommodation of national requirements. Actors have drawn on individual rights and the need of violence prevention as a strategy of public reasoning. These frames have opened up margins of negotiation regarding institutional conflicts between religious freedom and the prescription of surveillance. Very differently, a third case (Lower Saxony) has revealed a framing strategy that draws on group equality while pursuing top-down institutionalisation of Islam. The accommodation of intra-prison tension, in this case, has followed the reproduction of balances also valid for Christian communities. These framing strategies seem to be intimately linked to the respective position of the actors in their local contexts. While well-established Muslim actors in Lower Saxony who are close to official recognition as *Religionsgemeinschaft* raised their claim as part of their general ambition for group rights equality, less well-connected actors in the fragmented Muslim organizations’ landscapes in Berlin and Hessen (Wiesbaden) had to rely on alternative frames that would more easily open up space for a re-definition of the chaplain’s rights within the prison walls.

On this backdrop, the contribution offered by the present article is twofold: on the one hand Muslim prison chaplaincy constitutes a not yet well explored phenomenon in the German context. The empirical insights of this study join an increasing literature on Muslim chaplaincy in multiple European countries and could be further employed for cross-national comparison. In particular, the findings on how meso-level negotiations accommodate national legal frames and further impact the practice ‘on the ground’ (here exemplified through the right to secrecy) might subsequently be compared to the construction process of Muslim prison chaplaincy in other European countries.

On the other hand, this article has advocated the up to now rather unexplored view of subnational comparison in a federal state. While existing research on governance dynamics regarding religious claims has mostly taken a cross-national perspective thereby emphasising the differentiated impact of national models of diversity governance, subnational comparison allows for the observation of intra-national variations. It presents the advantage of taking into consideration different local sites and tensions inbuilt into national legal frameworks while holding constant the institutional context whose impacts are to be understood. This specific perspective brings to light what has not yet been fully developed in studies on chaplaincy and on diversity governance more broadly: the strategic use religious actors make of their institutional environment in order to negotiate their place in the public sphere and the variation of these strategies depending on the actor’s position (well-connected and legally recognized as *Religionsgemeinschaft* versus fragmented actor landscapes without legal status). Indeed, even though some publications on Muslim chaplaincy have pointed to locally ‘varying geometries’ of diversity accommodation (Béraud et al., 2013) – thereby emphasising the weight of individual entrepreneurs and perceptions – the articulation of diversity governance along federal lines in a legally highly codified regime shows more clearly the strategic construction of such ‘varying geometries’ by collective actors and individual entrepreneurs. Thus, while the findings of the present article illustrate the ‘dual mechanism’ that Bowen and his colleagues have pointed out regarding the mutual influence of national ideologies and concrete institutional practices, pragmatism, and boundaries, the examined subnational variation also extends this analytical framework by bringing to light the strategic role of actors depending on their social position and networks.

The comparison then ultimately demonstrates that neither the influence of national legal ideologies nor the institutional location of specific rules and practices are sufficient to fully account for the dynamics of religious diversity inclusion. Rather, both are mediated through a field of actors who combine and reshape their environment wherever spaces of negotiation emerge. Field-theoretical approaches to the governance of religious diversity taking into account power distribution and interests in a field facing permanent tension and negotiation of the rules of the game might in this perspective ultimately contribute to a better understanding of change in the governance of religion (Koenig, 2015; Michalowski, 2015; Thomas, 2004).

The exploration of subnational variation will also be illuminating for further comprehension of the articulation between the transnational, national and local level in the incorporation process of religious minorities. At the time being, a national conference of Muslim chaplaincy organised by the German *Islamkonferenz* is in preparation. Actors who experienced initial formalisation of Muslim chaplaincy at the regional level are invited and will share their insights at the national forum. In this perspective, the present article lays the ground for further analysis of the development of Muslim chaplaincy emergence and the interaction of national and regional levels. Indeed, how will national and regional fields of diversity governance be articulated in the ongoing institutionalisation of Muslim prison chaplaincy? And to what extent will regionally negotiated concepts resonate back to the national level and other regional sites? National negotiation and eventual transnational exchange about practices of diversity inclusion can only be fully understood if one takes into account struggles and varying accommodations coexisting in the national realm at the regional and local level.

## Footnotes

1 French scholars of sociology of institutions put very much in advance the fact that organisational settings are negotiated on a day-to-day basis by those investing these settings (see in particular the work of Jacques Lagroye and Michel Offerlé; (Lagroye & Offerlé, 2010)). Works on how ‘street level bureaucrats’ occupy the margins of action of public institutions point in a similar direction (Lipsky & Hill, 1997). New institutionalism scholars pointed not only to the importance of mutual influence between institutional settings (Friedland & Alford, 1991), but also to the role actors play in contention over the interpretation of institutions (Thomas, 2004).

2 Certain rights, such as the right to raise taxes only belong to those religious communities recognised as religious corporations.

3 Deutscher Bundestag: *Stand der rechtlichen Gleichstellung des Islam in Deutschland*. Antwort der Bundesregierung auf die Große Anfrage der Abgeordneten Josef Philip Winkler, Volker Beck (Köln), Renate Künast, Monika Lazar und der Fraktion BÜNDNIS 90/DIE GRÜNEN, Drucksache 16/5033, 18.04.2007. p. 62.

4 Interview, Ministry of Justice, Hessen, written questionnaire, February 2014.

5 Contract about Muslim Prison Chaplaincy in Lower Saxony: Vereinbarung zwischen dem Landesverband der Muslime in Niedersachsen e.V., Schura Niedersachsen, vertreten durch Herrn Avni Altiner, dem DITIB Landesverband der Islamische Religionsgemeinschaften Niedersachsen und Bremen e.V., vertreten durch Herrn Yilmaz Kilic und dem Niedersächsischen Justizministerium, vertreten durch Herrn Minister Bernd Busemann, 2012.

6 Together, the two umbrella organisations regroup almost 90% of Lower Saxony’s mosques. Both organisations started organising religious teaching in public schools and are involved in a dense cooperation network with state officials.

7
http://www.if-berlin.de/pressemitteilung-muslimische-vertreter-sagen-islamforum-ab.html.

8 Interview, Ministry of Justice, Hessen, written questionnaire, February 2014.

9 In total, 28 interviews have been conducted with 28 different interlocutors (some interlocutors were interviewed twice). Most of these interviews have been conducted between January and March 2014. Lower-Saxony: one practising Muslim chaplain, two Christian chaplains working in the prison of Hanover, five representatives of Muslim organisations, the official interlocutor at the Ministry for Justice for the contract about Muslim prison chaplaincy (interviewed twice). Berlin: the Muslim representative of the Muslim chaplaincy initiative (interviewed twice), two representatives of the Muslim organisation *lichtjugend*, the director of the Sehitlik Mosque who participated in the negotiation process, the representative of the round table of foreign prisoners, the representative of the Integration Council of Berlin, the official interlocutor at the Senate for Justice (interviewed twice), two interlocutors from the prison administration of Berlin Tegel. Hessen: two imams practising as Muslim prison chaplains (one of them interviewed twice), the directors of the prisons of Wiesbaden and Frankfurt, the representative of the Council of Integration of Wiesbaden, the representative of the Ministry of Justice of Hessen (written correspondence). Additionally: An expert lawyer (Dec. 2013), a Christian chaplain who published statements about Muslim prison chaplaincy

10 Five representatives of DITIB and Schura as well as the Ministry’s representative all started their report by recalling the decisive step made by Christian Wulff.

11 Vladi, Firouz: « Kooperation zwischen muslimischen Verbänden und der Landesregierung Niedersachsen. » In: Heinrich Böll Stiftung: *Muslimische Gemeinschaften zwischen Recht und Politik*, 2010, pp. 55–62.

12 Interview, representative Schura, Lower Saxony, 15.01.2014.

13 Interview, representative Schura, Lower Saxony, 27.01.2014.

14 Interview, representative of freiabonnements e.V., Berlin, 22.01.2014.

15 Lichtjugend, Islamische Föderation, DITIB, Muslimische Seelsorgetelefon, Haus der Weisheit, Initiative Berliner Muslime, Gemeinschaft Muslimischer Juristen

16 Interview, legal expert, Erfurt, 18.12.2013.

17 Interview, Official of the Ministry of Justice, Berlin, 26.07.2013.

18 Interview, representative of Berlin Sehitlik Mosque, Berlin, 05.02.2014.

19 Interview, representatives of prison administration, Berlin, 27.02.2014.

20 Interview, representative Berlin Sehitlik Mosque, Berlin, 05.02.2014.

21
http://www.rbb-online.de/politik/thema/fluechtlinge/berlin/2016/04/muslimische-gefaengnisseelsorge-berlin.html.

22 Landeshauptstadt Wiesbaden, “Vereinbarung zur gemeinsamen Förderung der Integration durch Zusammenarbeit (Integrationsvereinbarung”, 27.09.2007. http://www.wiesbaden.de/leben-in-wiesbaden/gesellschaft/auslaendische-buerger/content/integrationsvereinbarung.php.

23 In our interview he constantly pointed to the importance of the prevention work. He advocated the urgency of anti-radicalisation work in several articles published at the time when the fieldwork has been conducted. Meyer, Husamuddin: « Der Vollzugsalltag aus der Sicht der islamischen Seelsorge. » In: Hadmut Birgit Jung-Silberreis: 50 Jahre Jugendstrafvollzug in Wiesbaden –eine Festschrift. 1963–2013, Justizvollzugsanstalt Wiesbaden, 2013, pp. 239–245.; Meyer, Husamuddin: « Muslimische Gefangenenseelsorge. » In: Forum Strafvollzug. Zeitschrift für Strafvollzug und Straffälligenhilfe. Vol. 63, N°1, 2014, pp. 20–23. ; Topçu, Canan: “Glaubenslehre hinter Gittern.” Frankfurter Allgemeine Zeitung, 10.08.2014. http://www.faz.net/aktuell/rhein-main/gefaengnisseelsorge-glaubenslehre-hinter-gittern-13087256.html.

24 Both, the prison administration and the Muslim organisations underlined that the phenomenon of radicalisation in prison was at the time being no subject of preoccupation in Lower Saxony.

25 Request for the realisation of a pilot project in the *Land* Hessen, 23 November 2009. Document unpublished.

26 Interview, representative Council of Integration, Wiesbaden, 30.01.2014.

27 Meyer, Husamuddin: « Der Vollzugsalltag aus der Sicht der islamischen Seelsorge. » In: Hadmut Birgit Jung-Silberreis: 50 Jahre Jugendstrafvollzug in Wiesbaden –eine Festschrift. 1963–2013, Justizvollzugsanstalt Wiesbaden, 2013, pp. 239–245.; Meyer, Husamuddin: « Muslimische Gefangenenseelsorge. » In: Forum Strafvollzug. Zeitschrift für Strafvollzug und Straffälligenhilfe. Vol. 63, N°1, 2014, pp. 20–23.; Meyer, Husamuddin: « Muslimische Gefangenenseelsorge. » In: Forum Strafvollzug. Zeitschrift für Strafvollzug und Straffälligenhilfe. Vol. 63, N°1, 2014, pp. 20–23.

28 Interview, Ministry of Justice, Hessen, written questionnaire, February 2014.

29 Interview, prison director, Wiesbaden, 14.02.2014.

30 Interview, Protestant chaplain, Lower Saxony, 07.02.1014.

31 Rath, Christian: “Imame abhören erlaubt.” Taz.de, 11.03.2008. http://www.taz.de/!5185344/

32 Bundesgerichtshof, 15.04.2010, 4 StR 650/09.

33 Interview, representative Schura, Lower Saxony, 27.01.2014.

34 Contract about Muslim Prison Chaplaincy in Lower Saxony, p. 4.

35 Interview, Ministry of Justice, Lower Saxony, 17.01. and 21.02.2014.

36 The contract negotiated in Berlin has finally not been signed. Different interview partners recalled elements that figured in the contract-project.

37 Interview, two representatives of the prison administration, Berlin, 27.02.2014.

38 Interview, representative of the association of Muslim organisations for Muslim chaplaincy, Berlin, 24.02.2014.

39 Interviews conducted with several Muslim chaplains in the prison of Hannover and Berlin (Moabit).

40 Interview, Muslim chaplain, Berlin, 24.02.2014.

41 Interview, Husamuddin Meyer, Wiesbaden, 09.01.2014.

42 Interview, Protestant chaplain, Lower Saxony, 07.02.1014.
